# Molecular Epidemiology of and Risk Factors for Extensively Drug-Resistant *Klebsiella pneumoniae* Infections in Southwestern China: A Retrospective Study

**DOI:** 10.3389/fphar.2019.01307

**Published:** 2019-11-01

**Authors:** Xiaolang Tian, Changwu Huang, Xiaoli Ye, Hongyan Jiang, Rufang Zhang, Xiaofang Hu, Dongshuang Xu

**Affiliations:** Department of Clinical Laboratory, University of Chinese Academy of Sciences Chongqing Renji Hospital, Fifth People’s Hospital of Chongqing, Chongqing, China

**Keywords:** extensively drug-resistant *Klebsiella pneumoniae*, molecular epidemiology, MLST, risk factors, mortality

## Abstract

**Background:** The increasing prevalence of extensively drug-resistant *Klebsiella pneumoniae* (XDR-KP) poses a serious threat to clinical anti-infective treatment. This retrospective study assessed the molecular epidemiology of and risk factors for infections with XDR-KP to investigate the mechanism of drug resistance and the epidemiological characteristics.

**Methods:** A retrospective 1:2 case-control study was conducted at Chongqing Renji Affiliated Hospital of the Chinese Academy of Sciences University from January 2015 to December 2017. A total of 69 non-repetitive XDR-KP strains were collected. Patients infected with XDR-KP comprised the case group, and 138 matched patients with non-XDR-KP infection at the same site comprised the control group. The chi-square test and logistic regression were performed to evaluate the related risk factors. Molecular typing was performed by multilocus sequence typing (MLST). Potential resistance genes were detected by polymerase chain reaction (PCR) and sequencing. Predictors of 28-day mortality in patients with XDR-KP infection were also identified in our study.

**Results:** Only tigecycline and polymyxin B showed favorable *in vitro* drug sensitivity tests. These XDR-KP strains had a high prevalence rate (n = 66, 95.7%) of carbapenemase-related drug resistance genes. Among them, *KPC-2* was the most frequently detected gene (n = 52, 75.4%). Particularly, all of the isolates harbored multiple drug resistance genes. Epidemiological analysis showed that fifty-eight XDR-KP isolates were resistant strains with the ST-11 genotype. Multivariate logistic regression analysis showed that ICU admission (OR: 3.28, 95% CI: 1.66–6.49, *P* < 0.001), tracheal cannula (OR: 3.16, 95% CI: 1.48–6.76, *P* = 0.003), and carbapenem exposure (OR: 3.16, 95% CI: 1.25–7.98, *P* = 0.015) were independent risk factors for XDR-KP infection. Solid tumors (OR: 7.22, 95% CI: 1.84–28.34, *P* = 0.005) and septic shock (OR: 9.46, 95% CI: 2.00–44.72, *P* = 0.005) were independent risk factors for 28-day mortality from XDR-KP infection.

**Conclusion:** This study showed that XDR-KP isolates were highly resistant and exhibited clonal transmission. ST11 was the predominant epidemic type of XDR-KP producing *KPC-2* in Southwestern China. Physicians should be aware of these high-risk patients with notable predictive factors for XDR-KP infection. These findings may provide some recommendation for the diagnosis and treatment of patients infected with XDR-KP strains in Southwestern China.

## Introduction

*Klebsiella pneumoniae* (KP) is one of the most common pathogens of nosocomial infections that often cause severe or fatal infections (Blin et al., 2017; Liu et al., 2019). Carbapenems are currently the most effective beta-lactam antibiotics for the treatment of such severe bacterial infections. With the overuse of carbapenems in clinical treatment over the past decade, the emergence of carbapenem-resistant *K. pneumoniae* (CRKP) has become the main cause of the failure of clinical anti-infective treatment (Arabaghian et al., 2019; Barnes et al., 2019; Meng et al., 2019). It is worth noting that extensively drug-resistant *K. pneumoniae* (XDR-KP), which has a broader spectrum of drug resistance than CRKP, should be paid more attention. Because XDR-KP is resistant to most of the antibiotics currently used in the clinic and is only sensitive to tigecycline and polymyxin B, it further reduces the types of drugs available in the clinic, which poses a serious challenge to clinical anti-infective treatment (Du et al., 2016; Liu et al., 2017; Moradigaravand et al., 2017).

High morbidity and mortality are closely related to infection with XDR-KP, with a mortality rate of approximately 40–50% (Hoxha et al., 2016; Zheng et al., 2017). Currently, colistin is one of the few effective antibiotics in the treatment of XDR-KP infection, and the therapeutic options for infections are extremely limited. However, with the emergence of *mcr-1*-positive *Klebsiella pneumoniae*, which may lead to resistance to colistin, the treatment of XDR-KP infection is increasingly difficult (Rojas et al., 2017; Petrosillo et al., 2019). In addition, because carbapenem genes and other resistance genes can be transmitted frequently through mobile DNA elements, the increasing incidence of XDR-KP infection reported worldwide reflects a serious threat to public health and has even caused fatal outcomes in vulnerable patients. Therefore, it is necessary to explore the molecular epidemiology of and risk factors for infections with XDR-KP (de Campos et al., 2018; Li et al., 2018; Liu et al., 2019).

At present, there have been no reports on the molecular epidemiology and risk factors for XDR-KP infection in Southwestern China. In this retrospective study, we attempted to analyze the clinical characteristics, molecular epidemiology and risk factors for XDR-KP infection collected from Chongqing Renji Affiliated Hospital of the Chinese Academy of Sciences University to provide a theoretical basis for the clinical diagnosis, treatment and control of the spread of XDR-KP infection. Moreover, predictors for mortality in patients with XDR-KP infection were also identified in our study.

## Materials and Methods

### Study Design and Setting

This retrospective study was performed from January 2015 to December 2017 in the Chongqing Renji Affiliated Hospital of the Chinese Academy of Sciences University, a tertiary university hospital with a 1,800-bed academic medical centre located in Chongqing, Southwest China. Patients with XDR-KP infection were only sensitive to tigecycline or polymyxin B and resistant to all other drugs in this study. To assess for risk factors for XDR-KP infection, a retrospective 1:2 case-control study was performed. The case-control study groups in our analysis were defined as follows: the case group comprised patients with XDR-KP infection during hospitalization, and patients with non-XDR-KP infection at the same site comprised the control group. Patients admitted for <48 h and those with duplicate isolates were excluded.

### Identification and Drug Sensitivity of Bacteria

Bacterial cultures were processed in the clinical microbiology laboratory. All the XDR-KP isolates were identified using the VITEK 2 Compact system or the VITEK MS system (bioMérieux, Marcy l’Etoile, Lyon, France), and antimicrobial susceptibilities were determined *in vitro* using a VITEK 2 Compact AST-GN13 card (bioMérieux), which were used to test the antibiotic susceptibilities of all isolates to ampicillin (AMP), ampicillin/sulbactam (SAM), piperacillin (PIR), cefazolin (CFZ), ceftazidime (CAZ), ceftriaxone (CRO), cefepime (FEP), cefoxitin (FOX), aztreonam (AZT), gentamicin (GM), tobramycin (TOB), amikacin (AK), ciprofloxacin (CIP), and levofloxacin (LEV). Five antibiotics, including imipenem (IMP), meropenem (MEM), ertapenem (ETP), tigecycline (TGC) and polymyxin B (PB), were tested by the broth microdilution method following the criteria of the Clinical and Laboratory Standards Institute (CLSI, 2016). The minimal inhibitory concentrations (MICs) breakpoint for tigecycline was defined according to the European Committee on Antimicrobial Susceptibility Testing (EUCAST, 2017), while the others were interpreted according to CLSI protocols. *K. pneumoniae* ATCC700603 and *Escherichia coli* ATCC25922 were used as quality control strains for the antibiotic susceptibility tests.

### Identification of Resistance Genes

Crude DNA extracts were prepared by boiling the bacterial suspensions. This DNA was used as a template in polymerase chain reactions (PCR) to detect antibiotic resistance genes. Standard PCR conditions were used to amplify genes encoding carbapenemase-related genes (*blaKPC*, *blaNDM*, *blaVIM*, *blaIMP*, and *blaOXA*), extended spectrum β-lactamases (*blaTEM*, *blaSHV*, *blaCTX-M-1*, and *blaCTX-M-9*), plasmid-mediated AmpC (*blaEBC*, *blaDHA*, *blaCIT*, *blaCMY*, and *blaMOX*), quinolone resistance determinants (*qnrA*, *qnrB*, *qnrS*, and *acc(6’)-Ib-cr*), and aminoglycoside resistance determinants (*armA*, *rmtB*, and *acc(6’)-Ib*) using primers and PCR conditions described previously (Dallenne et al., 2010; Poirel et al.,2011; Abrar et al., 2019). The sequence of all primers used in this study was listed in **Table S1**. The PCR amplicons of carbapenemase, extended spectrum β-lactamases (ESBLs) and AmpC resistance genes were sequenced and the sequences were analyzed on the National Center for Biotechnology Information (NCBI) website.

### Data Collection and Definitions

After patients in both the case and control groups had been confirmed, relevant demographics and clinical data of the enrolled patients were collected from the electronic medical records and clinical microbiology laboratory databases. Survivor and nonsurvivor subgroups were compared to identify predictors of 28-day mortality.

#### Variables

Thirty-six clinical variables were applied in the analysis of the risk factors. The following parameters were used: (1) demographics (age, sex, hospital transfer, intensive care unit admission, and 28-day mortality); (2) baseline diseases and acquired infection (hypertension, diabetes, severe anemia, hypoproteinemia, hypokalemia, septic shock and solid tumors, respiratory, liver, gastrointestinal, cardiovascular, renal, and endocrine system diseases, as well as pulmonary, intra-abdominal, and urinary tract infection); (3) invasive procedures during the hospital stay (parenteral nutrition, mechanical ventilation, urinary catheter, tracheal cannula, drainage tube, previous surgery in the past 6 months, central venous catheter, and arterial catheter); and (4) antibiotic exposure within 3 months (cephalosporins, carbapenem, aminoglycosides, quinolones, tetracyclines, macrolides, metronidazole, and glycopeptides). These variables were analyzed to identify risk factors for XDR-KP infection.

#### Definitions

The following terms were defined prior to data analysis. Patients >65 years old were defined as elderly. Severe anemia was defined as a hemoglobin level <60 g/L. Hypoproteinemia was defined as serum total protein level <60 g/L or albumin level <25 g/L. Hypokalemia was defined as a serum potassium level <3.5 mmol/L. Septic shock was defined as sepsis associated with organ dysfunction and accompanied by persistent hypotension after volume replacement (Plante 2016; Howell and Davis, 2017). The source of infection was determined to be pneumonia, urinary tract infection (UTI), surgical site infection, intra-abdominal infection, or line-related infection using definitions from the US Centers for Disease Control and Prevention (CDC) (Kang et al., 2005).

### Multilocus Sequence Typing

Molecular typing was performed by multilocus sequence typing (MLST). The *K. pneumoniae* MLST scheme used internal fragments of the following seven housekeeping genes: beta-subunit of RNA polymerase (*rpoB*), glyceraldehyde 3-phosphate dehydrogenase (*gapA*), malate dehydrogenase (*mdh*), phosphoglucose isomerase (*pgi*), phosphorine E (*phoE*), translation initiation factor 2 (*infB*), and periplasmic energy transducer (*tonB*). The housekeeping genes primer sequence of *K. pneumoniae* was shown in **Table S2**. The PCR conditions were queried, and data were analyzed using the *K. pneumoniae* MLST website (http://bigsdb.web.pasteur.fr/klebsiella/klebsiella.html) (Joseph and Forsythe, 2017; Kiatsomphob et al., 2019; Shoaei et al., 2019).

### Sample Size Calculations and Statistical Analysis

Based on the report of the CHINET Antimicrobial Resistance Surveillance Program in 2015 (Fupin et al., 2016), we assumed that XDR-KP will comprise 2.2% of the cases and non-XDR-KP controls will comprise 14.1%. To determine a difference at the 0.05 significance level with 80% power, we estimated that we would need at least 66 XDR-KP vs. 132 non-XDR-KP control cases (EpiInfo, version 3.3.2).

All statistical analyses were performed using SPSS version 23.0 for Windows (SPSS, Inc., Chicago, IL, USA). Categorical variables were presented as frequencies and percentages. In the analysis of risk factors for XDR-KP infection and mortality, the univariate analysis was used for each variable. To identify the independent risk factors, variables with P < 0.10 in the univariate analysis were included in the logistic regression model for the multivariate analysis and analyzed using backward stepwise regression. The odds ratio (OR) and 95% confidence interval (CI) were also calculated to evaluate the strength of any association that emerged. For all statistical analyses, P < 0.05 indicated statistical significance.

## Results

### Bacterial Isolates

In this study, a total of 69 consecutive nonduplicate XDR-KP isolates were identified from January 2015 to December 2017 and investigated. Isolates originated from different anatomical sites: sputum (n = 21, 30.4%), urine (n = 17, 24.6%), blood (n = 9, 13.0%), bile (n = 7, 10.1%), secretions (n = 5, 7.2%), pus (n = 5, 7.2%), puncture fluid (n = 4, 5.8%), and cannula (n = 1, 1.4%). The majority of patients were in the intensive care unit (n = 23, 33.3%), followed by patients on the hepatobiliary surgical ward (n = 18, 26.1%), respiratory ward (n = 13, 18.8%), urinary surgery ward (n = 7, 10.1%), digestive medicine ward (n = 4, 5.8%), endocrine ward (n = 3, 4.3%) and obstetrics and gynecology ward (n = 1, 1.4%). A total of 138 patients with non-XDR-KP infection were randomly matched in a ratio of 1:2 as the control group. Therefore, 207 patients were included in the final study cohort.

### Antibiotic Susceptibility Test

As shown in **Table 1**, all 69 XDR-KP isolates were resistant to common clinical antibacterial drugs including carbapenems, by *in vitro* drug sensitivity tests but were only sensitive to tigecycline and polymyxin B (sensitivity rate 100%). The resistance rate of strains in the XDR-KP infection group was significantly higher than that in the control group, and the difference was statistically significant (P < 0.05).

**Table 1:** The antimicrobial susceptibility of extensively drug-resistant *Klebsiella pneumoniae* (XDR-KP) and non-XDR-KP.

Antibiotics	XDR-KP (n = 69)			non-XDR-KP (n = 138)			*P*-value
	S	I	R	S	I	R	
Ampicillin	0 (0)	0 (0)	69 (100)	15 (10.9)	4 (2.9)	119 (86.2)	**0.004**
Ampicillin/sulbactam	0 (0)	0 (0)	69 (100)	70 (50.8)	2 (1.4)	66 (47.8)	**<0.001**
Piperacillin	0 (0)	0 (0)	69 (100)	77 (55.8)	8 (5.8)	53 (38.4)	**<0.001**
Cefazolin	0 (0)	0 (0)	69 (100)	55 (39.9)	5 (3.6)	78 (56.5)	**<0.001**
Ceftriaxone	0 (0)	0 (0)	69 (100)	93 (67.4)	3 (2.2)	42 (30.4)	**<0.001**
Ceftazidime	0 (0)	0 (0)	69 (100)	94 (68.1)	4 (2.9)	40 (29.0)	**<0.001**
Cefepime	0 (0)	0 (0)	69 (100)	104 (75.4)	8 (5.8)	26 (18.8)	**<0.001**
Cefoxitin	0 (0)	0 (0)	69 (100)	101 (73.2)	5 (3.6)	32 (23.2)	**<0.001**
Aztreonam	0 (0)	0 (0)	69 (100)	68 (49.2)	11 (8.0)	59 (42.8)	**<0.001**
Ciprofloxacin	0 (0)	0 (0)	69 (100)	87 (63.0)	0 (0.0)	51 (37.0)	**<0.001**
Levofloxacin	0 (0)	0 (0)	69 (100)	91 (65.9)	4 (2.9)	43 (31.2)	**<0.001**
Gentamicin	0 (0)	0 (0)	69 (100)	90 (65.2)	7 (5.1)	41 (29.7)	**<0.001**
Tobramycin	0 (0)	0 (0)	69 (100)	104 (75.4)	8 (5.8)	26 (18.8)	**<0.001**
Amikacin	0 (0)	0 (0)	69 (100)	118 (85.5)	5 (3.6)	15 (10.9)	**<0.001**
Ertapenem	0 (0)	0 (0)	69 (100)	126 (91.4)	2 (1.4)	10 (7.2)	**<0.001**
Imipenem	0 (0)	0 (0)	69 (100)	129 (93.5)	0 (0)	9 (6.5)	**<0.001**
Meropenem	0 (0)	0 (0)	69 (100)	129 (93.5)	0 (0)	9 (6.5)	**<0.001**
Tigecycline	69 (100)	0 (0)	0 (0)	138 (100)	0 (0)	0 (0)	**–**
Polymyxin B	69 (100)	0 (0)	0 (0)	138 (100)	0 (0)	0 (0)	**–**

Values are presented as n (%), unless otherwise noted. S, susceptible; I, intermediate-resistant; R, resistant. “–” indicates data not available. Bold face indicates values that are significant (P < 0.05).

### Detection of Drug Resistance Genes and ST Types

The distribution of resistance genes and ST types are listed in **Table 2**. Overall, these XDR-KP isolates had a high prevalence rate of carbapenemase-related resistance genes (n = 66, 95.7%), and only three strains were not determined to have carbapenemase-related resistance genes. Among them, the *KPC-2* gene was mainly carried (n = 52, 75.4%), followed by the *NDM* (n = 21, 30.4%) and *IMP* (n = 5, 7.2%) genes. The *OXA-48* gene was also detected in three XDR-KP strains in our study. Notably, several isolates were determined to carry two carbapenemase-related resistance genes at the same time (n = 15, 21.7%). In addition, all the XDR-KP isolates contained multiple drug resistance genes, including extended spectrum β-lactamases (ESBLs), AmpC β-lactamases, quinolone resistance determinants, and aminoglycoside resistance determinants. Among them, *CTX-M-9* (n = 38, 55.1%), *DHA* (n = 29, 42.0%), *qnrS* (n = 40, 57.9%) and *acc (6’)-Ib* (n = 43, 62.3%) were the most frequently detected genes among these drug resistance genes, respectively.

**Table 2:** Distribution of resistance genes and MLST types of extensively drug-resistant *Klebsiella pneumoniae* (XDR-KP).

Microorganism(no. of strains)	Resistance genes and MLST types (no. of strains)					ST types
	Carbapenemase resistance genes	ESBL resistance genes	AmpC resistance genes	Quinolone resistance determinants	Aminoglycoside resistance determinants	
XDR-KP (69)	*KPC-2* (52)	*CTX-M-1* (17)	*DHA* (29)	*qnrA* (12)	*armA* (3)	ST11 (58)
	*NDM-1* (16)	*CTX-M-9* (38)	*CMY* (14)	*qnrB* (19)	*rmtB* (36)	ST20 (5)
	*NDM-5* (5)	*TEM-1* (28)	*MOX* (16)	*qnrS* (40)	*acc(6’)-Ib* (43)	ST45 (1)
	*IMP-4* (4)	*SHV-2* (6)		*acc(6’)-Ib-cr* (28)		ST592 (2)
	*IMP-8* (1)	*SHV-11* (10)				ST661 (2)
	*OXA-48* (3)	*SHV-12* (18)				ST751 (1)

MLST, multilocus sequence typing; ESBL, extended-spectrum β-lactamase.

Drug resistance genes can be transmitted horizontally among bacteria through mobile DNA elements, such as plasmids and integron genes. Molecular typing can assist in the analysis of transmission pathways between bacterial pathogens. The results of MLST showed that six different ST types were identified, of which ST11 was the most predominant epidemic type (n = 58, 84.1%). Perhaps more significantly, all strains of the ST11 type harbored the *KPC-2* gene, which indicated that the *KPC-2* gene was closely related to the prevalence of the XDR-KP strain.

### Risk Factor Analysis of XDR-KP Infection

Univariate analysis of the demographics, baseline diseases, acquired infection, invasive procedures, and antibiotic exposure was performed in the case-control groups. The following factors were most frequently associated with the development of XDR-KP infection: ICU admission, transferring from another hospital, respiratory diseases, pulmonary infection, tracheal cannula, and exposure to carbapenem and glycopeptide antibiotics (**Table 3**).

**Table 3:** Univariate analysis of risk factors for infection with extensively drug-resistant *Klebsiella pneumoniae* (XDR-KP) and non-XDR-KP.

Variables	XDR-KP infection (n = 69)	Control group (n = 138)	OR (95% CI)	*P-value*
**Demographics**				
Male gender	37 (53.6)	72 (52.2)	1.06 (0.59–1.89)	0.844
Elderly	31 (44.9)	58 (42.0)	1.13 (0.63–2.02)	0.691
ICU admission	37 (53.6)	33 (23.9)	3.68 (1.99–6.79)	**<0.001**
Transferring from another hospital	26 (37.7)	33 (23.9)	1.92 (1.03–3.59)	**0.039**
**Baseline diseases and acquired infection**				
Hypertension	25 (36.2)	36 (26.1)	1.61 (0.87–2.99)	0.131
Diabetes	18 (26.1)	27 (19.6)	1.45 (0.73–2.87)	0.284
Hypoproteinemia	18 (26.1)	32 (23.2)	1.16 (0.60–2.27)	0.646
Hypokalemia	15 (21.7)	18 (13.0)	1.85 (0.87–3.95)	0.107
Severe anemia	4 (5.8)	9 (6.5)	0.88 (0.26–2.97)	0.839
Septic shock	18 (26.1)	22 (15.9)	1.86 (0.92–3.77)	0.081
Solid tumors	23 (33.3)	38 (27.5)	1.32 (0.71–2.46)	0.388
Respiratory diseases	25 (36.2)	31 (22.5)	1.96 (1.04–3.69)	**0.036**
Liver diseases	19 (27.5)	34 (24.6)	1.16 (0.60–2.24)	0.652
Gastrointestinal diseases	15 (21.7)	28 (20.3)	1.09 (0.54–2.21)	0.809
Cardiovascular diseases	10 (14.5)	12 (8.7)	1.78 (0.73–4.35)	0.202
Renal diseases	15 (21.7)	35 (25.4)	0.82 (0.41–1.63)	0.566
Endocrine system diseases	6 (8.7)	20 (14.5)	0.56 (0.21–1.47)	0.235
Pulmonary infection	16 (23.2)	17 (12.3)	2.15 (1.01–4.57)	**0.044**
Intra-abdominal infection	9 (13.0)	16 (11.6)	1.14 (0.48–2.74)	0.763
Urinary tract infection	10 (14.5)	23 (16.7)	0.85 (0.38–1.90)	0.687
**Invasive procedures during hospital stay**				
Parenteral nutrition	11 (15.9)	15 (10.9)	1.56 (0.67–3.59)	0.299
Mechanical ventilation	16 (23.2)	19 (13.8)	1.89 (0.90–3.96)	0.088
Urinary catheter	29 (42.0)	51 (37.0)	1.24 (0.69–2.23)	0.481
Tracheal cannula	26 (37.7)	19 (13.8)	3.79 (1.91–7.53)	**<0.001**
Drainage tube	8 (11.6)	23 (16.7)	0.66 (0.28–1.55)	0.335
Previous surgery in the past 6 months	24 (34.8)	38 (27.5)	1.40 (0.76–2.61)	0.283
Central venous catheter	10 (14.5)	18 (13.0)	1.13 (0.49–2.60)	0.774
Arterial catheter	9 (13.0)	25 (18.1)	0.68 (0.29–1.55)	0.353
**Antibiotic exposure within 3 months**				
Cephalosporins	26 (37.7)	48 (34.8)	1.13 (0.62–2.06)	0.682
Carbapenem	21 (30.4)	14 (10.1)	3.88 (1.82–8.23)	**<0.001**
Aminoglycosides	6 (8.7)	20 (14.5)	0.56 (0.22–1.47)	0.235
Quinolones	12 (17.4)	28 (20.3)	0.83 (0.39–1.75)	0.619
Tetracyclines	4 (5.8)	6 (4.3)	1.35 (0.37–4.97)	0.647
Macrolides	5 (7.2)	8 (5.8)	1.27 (0.39–4.04)	0.685
Metronidazole	18 (26.1)	25 (18.1)	1.59 (0.80–3.18)	0.183
Glycopeptides	12 (17.4)	11 (8.0)	2.43 (1.01–5.84)	**0.042**

Values are presented as n (%), unless otherwise noted. Bold face indicates values that are significant (P < 0.05). “–” indicates data not available.

ICU, intensive care unit.

### Multivariate Analysis of XDR-KP Infection

The results of the logistic regression analysis of XDR-KP infection are shown in **Table 4**: ICU admission (OR: 3.28, 95% CI: 1.66–6.49, *P* < 0.001), tracheal cannula (OR: 3.16, 95% CI: 1.48–6.76, *P* = 0.003), and carbapenem exposure (OR: 3.16, 95% CI: 1.25–7.98, *P* = 0.015) were identified as independent risk factors for infection with XDR-KP when compared with the control group.

**Table 4:** Multivariate analysis of risk factors for infection with extensively drug-resistant *Klebsiella pneumoniae* (XDR-KP) and non-XDR-KP.

Variables	B	S.E	Wals	OR	95% CI	*P-value*
ICU admission	1.19	0.35	11.60	3.28	1.66–6.49	**<0.001**
Tracheal cannula	1.15	0.39	8.84	3.16	1.48–6.76	**0.003**
Carbapenem exposure	1.15	0.47	5.90	3.16	1.25–7.98	**0.015**

Bold face indicates values that are significant (P < 0.05). ICU, intensive care unit.

### Risk Factors for 28-Day Mortality

At 28 days after infection onset, 21 of the 69 patients we investigated had died. Our study highlights the high mortality associated with XDR-KP infection, as shown in **Table 5**. The univariate analysis revealed that the presence of solid tumors, septic shock, and a tracheal cannula resulted in significant differences between the survivor and nonsurvivor groups. Multivariate logistic regression analysis showed that the predictors independently associated with 28-day mortality were solid tumors (OR: 7.22, 95% CI: 1.84–28.34, *P* = 0.005), and septic shock (OR: 9.46, 95% CI: 2.00–44.72, *P* = 0.005).

**Table 5:** Risk factors associated with 28-day mortality.

Variables	Nonsurvivors (n = 21)	Survivors (n = 48)	OR (95% CI)	*P*-value	OR (95% CI)	*P*-value
Elderly	10 (47.6)	21 (43.8)	1.17 (0.42–3.27)	0.766	**–**	**–**
ICU admission	9 (42.9)	28 (58.3)	0.54 (0.19–1.51)	0.236	**–**	**–**
Solid tumors	14 (66.7)	9 (18.8)	8.67 (2.71–27.68)	**<0.001**	7.22 (1.84–28.34)	**0.005**
Respiratory diseases	9 (42.9)	16 (33.3)	1.50 (0.52–4.30)	0.449	**–**	**–**
Digestive system disease	7 (33.3)	12 (25.0)	1.50 (0.49–4.59)	0.476	**–**	**–**
Urinary System Diseases	3 (14.3)	12 (25.0)	0.50 (0.13–1.99)	0.321	**–**	**–**
Cardiovascular diseases	4 (19.0)	6 (12.5)	1.65 (0.41–6.58)	0.477	**–**	**–**
Hypoproteinemia	8 (38.1)	10 (20.8)	2.34 (0.76–7.19)	0.133	**–**	**–**
Hypokalemia	7 (33.3)	8 (16.7)	2.50 (0.77–8.16)	0.122	**–**	**–**
Septic shock	11 (52.4)	7 (14.6)	6.44 (1.99–20.82)	**<0.001**	9.46 (2.00–44.72)	**0.005**
Parenteral nutrition	3 (14.3)	8 (16.7)	0.83 (0.20–3.51)	0.804	**–**	**–**
Tracheal cannula	12 (57.1)	14 (29.2)	3.24 (1.12–9.39)	**0.027**	**–**	**–**
Drainage tube	2 (9.5)	6 (12.5)	0.74 (0.14–3.99)	0.722	**–**	**–**
Previous surgery in the past 6 months	6 (28.6)	18 (37.5)	0.67 (0.22–2.03)	0.474	**–**	**–**
Central venous catheter	3 (14.3)	7 (14.6)	0.98 (0.23–4.21)	0.974	**–**	**–**
Arterial catheter	3 (14.3)	6 (12.5)	1.17 (0.26–5.19)	0.839	**–**	**–**
Mechanical ventilation	8 (38.1)	8 (16.7)	4.39 (1.35–14.28)	0.052	**–**	**–**
Cephalosporins	9 (42.9)	17 (35.4)	1.37 (0.48–3.89)	0.557	**–**	**–**
Carbapenem	7 (33.3)	14 (29.2)	1.21 (0.40–3.65)	0.729	**–**	**–**
Aminoglycosides	2 (9.5)	4 (8.3)	1.16 (0.20–6.87)	0.872	**–**	**–**
Quinolones	4 (19.0)	8 (16.7)	1.18 (0.31–4.44)	0.810	**–**	**–**
Macrolides	1 (4.8)	4 (8.3)	0.55 (0.06–5.24)	0.599	**–**	**–**

Values are presented as n (%), unless otherwise noted. Bold face indicates values that are significant (P < 0.05). “–” indicates data not available.

ICU, intensive care unit.

## Discussion

At present, the emergence and dissemination of the XDR-KP strain is a major worldwide source and shuttle for antibiotic resistance. The increasing prevalence and global spread of these clinically extensively drug-resistant strains gravely threatens public health (Liu et al., 2017). According to the global epidemic trend of XDR-KP strains, the detection rate of XDR-KP strains in the northeastern United States, Italy, Greece, Israel and other European countries was relatively high, and there were even reports of local epidemic outbreaks in some areas (Zhou et al., 2015; Gu et al., 2018). However, molecular epidemiological characteristics differ from region to region (Garg et al., 2019; Kim et al., 2019). To our knowledge, there have been no reports on the epidemiology of and risk factors for XDR-KP infection in Southwestern China. This retrospective study was the first to systematically analyze the clinical characteristics, molecular epidemiology, risk factors of XDR-KP infection and the predictors of mortality.

For the drug sensitivity of XDR-KP strains, only tigecycline and polymyxin B still showed favorable *in vitro* drug sensitivity tests, and they were the most sensitive antibiotics for XDR-KP infection in the present study (sensitivity rate 100%). Currently, tigecycline and polymyxin B are considered to be the last line of defense against XDR-KP infection (Pragasam et al., 2016; Braun et al., 2018; Alves et al., 2019). However, increasing resistance is reported worldwide, in particular due to the plasmid-encoded protein *mcr-1* present in pathogens such as *Escherichia coli* and *Klebsiella pneumoniae*, which further increases the difficulty of anti-infective treatment (Zhao et al., 2017; Shankar et al., 2019). Focusing on the molecular detection and genetic characterization of XDR-KP strains, we found that all the XDR-KP isolates contained multiple drug resistance genes, suggesting that the wide resistome of strains was related to the plentiful chromosomal and plasmid-encoded antibiotic resistance genes. The data revealed that the emergence of carbapenemase genes and ESBLs was the primary resistance mechanism. Among them, *KPC-2* and *CTX-M-9* group genes were the most prevalent in this study and were reported to be the most easily transmitted horizontally in KP (Fuga et al., 2019; Perez, 2019; Vargas et al., 2019). In addition, *DHA* was reported to be the most common gene in KP producing AmpC β-lactamases in many studies, which was consistent with our study (Akata et al., 2019; Kazemian et al., 2019). Notably, under antibiotic selective pressure, the pathogen continuously accumulates multiple drug resistance genes by acquiring plasmids and transferable genetic elements, ultimately leading to XDR strains harboring a ‘super resistome’ (Navon-Venezia et al., 2017).

MLST could be used to understand the epidemiology at a large scale in local outbreak situations. In the current study, ST11 was the most predominant epidemic type among the six different ST types identified, concordant with the report that ST11 was the major sequence type in Mainland China (Fu et al., 2019; Yu et al., 2019), and different from the international hyper-epidemic lineage ST258. Previous studies have shown that the hyperepidemic clonal complex of multidrug-resistant *K. pneumoniae* in ST258 clone may be spreading in the north-eastern region of Hungary (Toth et al., 2010), which were different from our results. Interestingly, only one of the seven housekeeping genes between ST11 (*gapA*, *infB*, *mdh*, *pgi*, *phoE*, *rpoB*, *tonB*: 3, 3, 1, 1, 1, 1, 4) and ST258 (*gapA*, *infB*, *mdh*, *pgi*, *phoE*, *rpoB*, *tonB*: 3, 3, 1, 1, 1, 1, 79) was different, suggesting that they were closely related to each other (Yu et al., 2018). Notably, all strains of ST11 XDR-KP isolates harbored the *KPC-2* gene in our study, suggesting that the *KPC-2* gene was involved in the epidemic dissemination of XDR-KP strains, which has frequently been reported worldwide in the interspecific transmission and dissemination of *Enterobacteriaceae* (Gao et al., 2019). If not effectively controlled, the ST11 clone could possibly spread widely in the future and even lead to the outbreak of XDR-KP strains.

Univariate analyses revealed that ICU admission, transferring from another hospital, respiratory diseases, pulmonary infection, tracheal cannula and exposure to carbapenem and glycopeptide antibiotics were strongly correlated with XDR-KP infection, which has also been identified by other studies. In addition, ICU admission, tracheal cannula, and carbapenem exposure were identified as independent risk factors for infection with XDR-KP in our study. Moreover, these ICU patients with concomitant infections had more severe underlying diseases and lower immunity, which might have made them more vulnerable to acquiring XDR-KP infection, and invasive procedures such as tracheal cannula and mechanical ventilation would have increased the possibility of drug-resistant bacteria adhesion, which ultimately led to infection with XDR-KP strains (Kiddee et al., 2019; Li et al., 2019). In addition, the selection pressure of antibiotics played an important role in the development of multiple drug resistance of bacteria. It was generally believed that under the pressure of carbapenem, susceptible strains were inhibited or killed, leading to the proliferation of many drug-resistant strains as dominant bacteria. Moreover, drug-resistant genes carried by drug-resistant bacteria could have been transmitted horizontally to susceptible strains by the acquisition of plasmids and transferable genetic elements, thus causing more serious epidemics of drug-resistant bacteria (Lee et al., 2013; Lopez-Camacho et al., 2014; Zhang et al., 2018). Therefore, we should select antibiotics according to drug sensitivity test results, and the abuse of carbapenem antibiotics should be avoided to reduce the production of XDR-KP strains.

Our study highlights the high mortality associated with XDR-KP infection. We found that solid tumors and septic shock were independent risk factors for death caused by XDR-KP infection. Most cancer patients usually have low immunity after radiotherapy or chemotherapy. After infection with “superbacteria” such as XDR-KP, these patients often received inappropriate initial antimicrobial treatment, which might damage prognosis and increase mortality. In addition, drug-resistant bacteria often cause serious infections, resulting in bacteremia or even sepsis in patients, causing septic shock that leads to multiple organ failure or even death (Falcone et al., 2016; Christodoulou et al., 2017; Machuca et al., 2017). Therefore, the surveillance of cancer and septic shock patients infected with XDR-KP should be strengthened to reduce their mortality.

### Limitations

Given that this was a retrospective, case-control study, it is not possible to determine the absolute risk associated with the identified risk factors. In addition, this retrospective study was conducted at a single medical centre, and our sample size was relatively small, which might have led to errors in statistical analysis and the omission of some other risk factors.

## Conclusions

This retrospective study was the first to systematically analyze the clinical characteristics, molecular epidemiology, and risk factors of XDR-KP infection and the predictors of mortality. Our findings showed that ST11 was the predominant epidemic type of XDR-KP producing *KPC-2* in Southwestern China. In addition, ICU admission, tracheal cannula, and carbapenem exposure were identified as independent risk factors for infection with XDR-KP in this study. Moreover, another important finding of this study was that solid tumors and septic shock were independent risk factors for death caused by XDR-KP infection. Therefore, physicians should be aware of these high-risk patients with notable predictive factors for XDR-KP infection to reduce their mortality rate.

## Data Availability Statement

The raw data supporting the conclusions of this manuscript will be made available by the authors, without undue reservation, to any qualified researcher.

## Ethics Statement

The study was approved by the Ethics Committee of Renji Hospital, Chinese Academy of Sciences University, Chongqing, China. The ethics committee waived the need for written informed consent provided by participants, and no additional informed consent was required.

## Author Contributions

XY, HJ, and RZ performed experiments. CH and XH conceived the study and analyzed the results. XT and DX supervised the study and prepared the manuscript. All authors read and approved the final paper.

## Funding

This study was supported in part by the National Natural Science Foundation of China (grant numbers 81471992 and 81772239), and the Natural Science Foundation of Chongqing (grant number 2019msxmX0568).

## Conflict of Interest

The authors declare that the research was conducted in the absence of any commercial or financial relationships that could be construed as a potential conflict of interest.
