# Supplementary material for: Molecular Epidemiology of and Risk Factors for Extensively Drug-Resistant Klebsiella pneumoniae Infections in Southwestern China: A Retrospective Study
Source: Front Pharmacol. 2019 Nov 1;10:1307. doi: 10.3389/fphar.2019.01307 (PMC6838015; doi:10.3389/fphar.2019.01307)
Supplement: Supplementary file 1 [file DataSheet_1.docx]

**Table S1** Primers used for ampliﬁcation

| **Gene** | **Sequence (5***'***-3***'***)** | **Amplicon size (bp)** | **Anneal tep (℃)** |
| --- | --- | --- | --- |
| *blaKPC* | F: ATGTCACTGTATCGCCGTCT  R: TTTTCAGAGCCTTACTGCCC | 892 | 52 |
| *blaNDM* | F: CAGCACACTTCCTATCTC  R: CCGCAACCATCCCCTCTT | 292 | 55 |
| *blaVIM* | F: TTATGGAGCAGCAACGATGT  R: CAAAAGTCCCGCTCCAACGA | 920 | 52 |
| *blaIMP* | F: CATGGTTTGGTTGTTCTTGT  R: ATAATTTAGCGGACTTTGGC | 488 | 55 |
| *blaOXA-48* | F: TTGGTGGCATCGATTATCGG  R: GAGCACTTCTTTTGTGATGGC | 438 | 55 |
| *blaTEM* | F: GTGCGCGGAACCCCTATT  R: TTACCAATGCTTAATCAGTGAGGC | 919 | 60 |
| *blaSHV* | F: CTTTACTCGCCTTTATCGGC  R: TTACCGACCGGCATCTTTCC | 1031 | 60 |
| *blaCTX-M-1* | F: AAGACTGGGTGTGGCATTGA  R: AGGCTGGGTGAAGTAAGTGA | 670 | 55 |
| *blaCTX-M-9* | F: GCTTTATGCGCAGACGAGTG  R: GCCAGATCACCGCAATATCA | 686 | 55 |
| *blaEBC* | F: CGGTAAAGCCGATGTTGCG  R: AGCCTAACCCCTGATACA | 683 | 54 |
| *blaDHA* | F: TGATGGCACAGCAGGATATTC  R: GCTTTGACTCTTTCGGTATTCG | 997 | 55 |
| *blaCIT* | F: CGAAGAGGCAATGACCAGAC  R: ACGGACAGGGTTAGGATAGY | 538 | 55 |
| *blaCMY* | F: TGGCCAGAACTACAGGCAAA  R: TTTCTCCTGAACGTGGCTGGC | 462 | 55 |
| *blaMOX* | F: GCAACAACGACAATCCATCCT  R: GGGATAGGCGTAACTCTCCCAA | 895 | 54 |
| *armA* | F: CCGAAATGACAGTTCCTATC  R: GAAAATGAGTGCCTTGGAGG | 846 | 55 |
| *rmtB* | F: ATGAACATCAACGATGCCCT  R: CCTTCTGATTGGCTTATCCA | 769 | 55 |
| *acc(6')-Ib* | F: TATGAGTGGCTAAATCGAT  R: CCCGCTTTCTCGTAGCA | 395 | 55 |
| *qnrA* | F: ATTTCTCACGCCAGGATTTG  R: GAGATTGGCATTGCTCCAGT | 413 | 53 |
| *qnrB* | F: GATCGTGAAAGCCAGAAAGG  R: ACGATGCCTGGTAGTTGTCC | 469 | 53 |
| *qnrC* | F: GGGTTGTACATTTATTGAATC  R: TCCACTTTACGAGGTTCT | 447 | 47 |
| *qnrD* | F: CGAGATCAATTTACGGGGAATA  R: AACAAGCTGAAGCGCCTG | 582 | 54 |
| *qnrS* | F: GCAAGTTCATTGAACAGGGT  R: TCTAAACCGTCGAGTTCGGCG | 428 | 58 |
| *aac(6’)-Ib-cr* | F: ATATGCGGATCCAATGAGCAACGCAA  R: ATAGCGAATTCTTAGGCATCACTGCG | 544 | 55 |

Table S2 House-keeping genes primer sequence of *K. pneumoniae*

| **Gene** | **Primer pair sequences** | **Product length** |
| --- | --- | --- |
| *rpoB* | F: GGCGAAATGGCWGAGAACCA R: GAGTCTTCGAAGTTGTAACC | 252–280  973–1000 |
| *gapA* | F: TGAAATATGACTCCACTCACGG R: CTTCAGAAGCGGCTTTGATGGC | 513–490  1211–1308 |
| *mdh* | F: CCCAACTCGCTTCAGGTTCAG R: CCGTTTTTCCCCAGCAGCAG | 163–190  862–1095 |
| *pgi* | F: GAGAAAAACCTGCCTGTACTGCTGGC R: CGCGCCACGCTTTATAGCGGTTAAT | 1142–1269  2459–2686 |
| *phoE* | F: ACCTACCGCAACACCGACTTCTTC R: TGATCAGAACTGGTAGGTGAT | 76–89  553–570 |
| *infB* | F: CTCGCTGCTGGACTATATTCG R: CGCTTTCAGCTCAAGAACTTC | 27–64  705–732 |
| *tonB* | F: CTTTATACCTCGGTACATCAGGTT R: ATTCGCCGGCTGRGCRGAGAG | 212–270  952–1033 |

**Note:** PCR amplification is performed at an annealing temperature of 50°C for all genes except for gapA (60°C) and tonB (45°C).
